# Stress granules: functions and mechanisms in cancer

**DOI:** 10.1186/s13578-023-01030-6

**Published:** 2023-05-13

**Authors:** Huan Zhou, Jing Luo, Kelin Mou, Lin Peng, Xiaoyue Li, Yulin Lei, Jianmei Wang, Sheng Lin, Yuhao Luo, Li Xiang

**Affiliations:** 1 Department of Oncology, The Affiliated Hospital of Southwest Medical University, Luzhou, China; 2 Department of Cardiology, The Affiliated Hospital of Southwest Medical University, Luzhou, China; 3 Department of Bone and Joint Surgery, The Affiliated Hospital of Southwest Medical University, Luzhou, China; 4 Department of Pathology, The Affiliated Hospital of Southwest Medical University, Luzhou, China; 5 Nuclear Medicine and Molecular Imaging Key Laboratory of Sichuan Province, Luzhou, China

**Keywords:** Stress granules, Signaling pathway, Therapy resistance, Immunotherapy, G3BP1/2

## Abstract

Stress granules (SGs) are non-enveloped structures formed primarily via protein and RNA aggregation under various stress conditions, including hypoxia and viral infection, as well as oxidative, osmotic, and heat-shock stress. SGs assembly is a highly conserved cellular strategy to reduce stress-related damage and promote cell survival. At present, the composition and dynamics of SGs are well understood; however, data on the functions and related mechanisms of SGs are limited. In recent years, SGs have continued to attract attention as emerging players in cancer research. Intriguingly, SGs regulate the biological behavior of tumors by participating in various tumor-associated signaling pathways, including cell proliferation, apoptosis, invasion and metastasis, chemotherapy resistance, radiotherapy resistance, and immune escape. This review discusses the roles and mechanisms of SGs in tumors and suggests novel directions for cancer treatment.

## Introduction

Cancer incidence and mortality remain high, which severely threatens human health and quality of life [1, 2]. While advances in treatment have improved the outcomes and overall quality of life in cancer patients, metastasis and therapy resistance remain major challenges associated with poor prognosis [3, 4]. Notably, previous studies have confirmed the involvement of stress granules (SGs), non-enveloped structures formed primarily via protein and RNA aggregation under various stress conditions, in cancer development and progression [5–7], and demonstrated their functions and mechanisms in cancer [6, 8, 9].

Understanding the SGs formation mechanism in tumor cell is essential for targeting SGs to improve tumor treatment efficacy. However, in addition to stress induction, the maladjustment of certain signaling pathways and gene mutations also contribute to the formation of SGs in tumor cell [5]. In this review, we conducted a systematic review of the inductions, disintegrations, functions, and molecular mechanisms of SGs in cancer. Meanwhile, we also discussed the clinical applications of SGs as novel therapeutic strategies for overcoming therapy resistance in cancer management.

### Basic molecular mechanism of SGs

#### The components of SGs

**Fig. 1:**
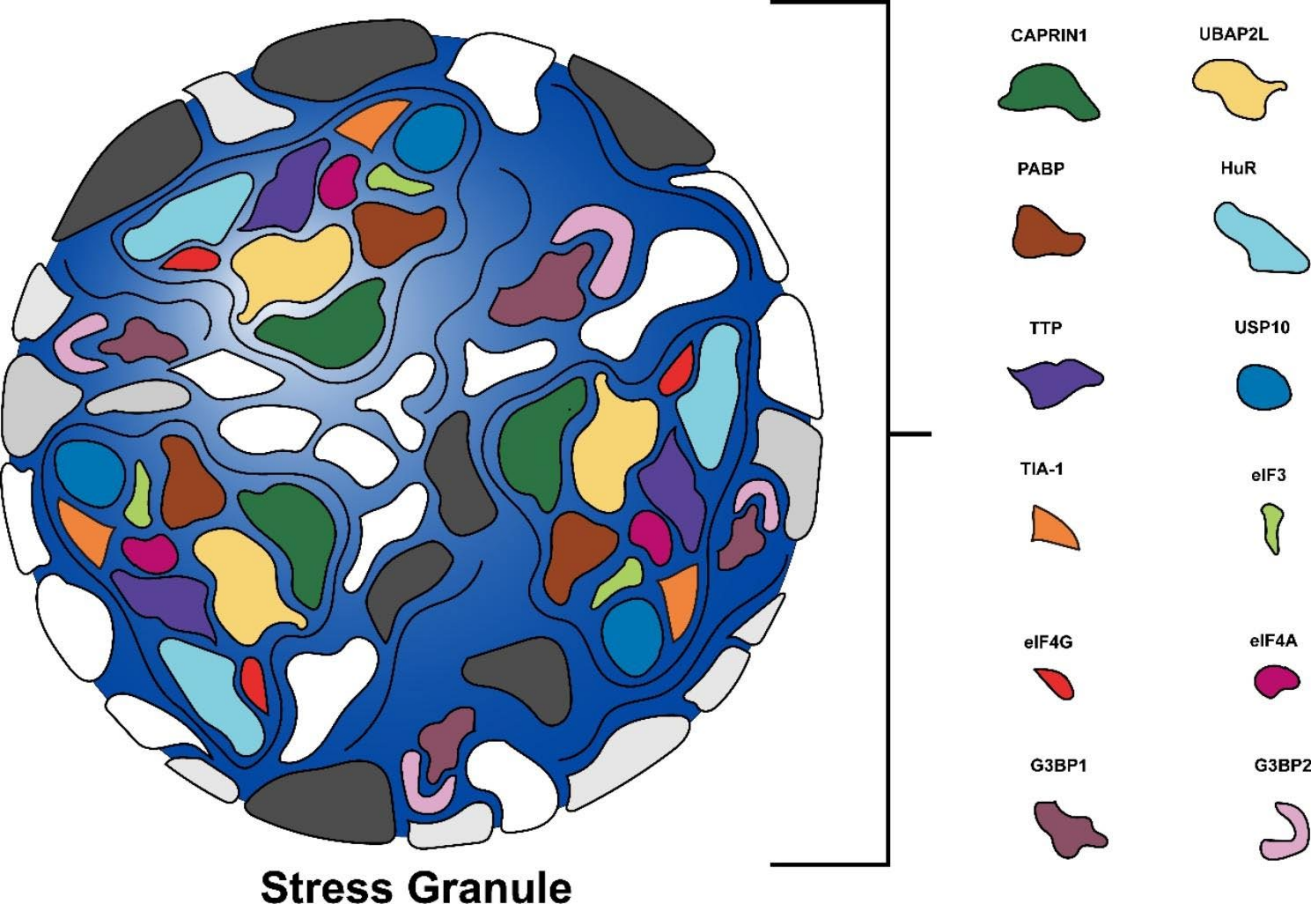
**Components of SGs.** SGs are composed of various translation initiation factors, 40 S ribosomal subunits, and non-translating mRNAs, as well as both RNA binding and non-RNA binding proteins

SGs constitute a prominent type of ribonucleoprotein (RNP) particle—a universal feature of eukaryotic cell located primarily in the cytoplasm [10], and consist of a stable core structure and dynamic shell [11]. SGs also contain various translation initiation factors, 40 S ribosomal subunits, and non-translating mRNAs, as well as both RNA binding and non-RNA binding proteins [12–16] (Fig. 1). These translation initiation factors include eukaryotic initiation factor 3 (eIF3), eukaryotic initiation factor 4G (eIF4G), and eukaryotic initiation factor 4 A (eIF4A). The associated RNA binding proteins (RBPs) include Ras-GAP SH3 domain binding protein (G3BP), poly(A)-binding protein (PABP), cell cycle associated protein 1 (CAPRIN1), ubiquitin associated protein 2 like (UBAP2L), human antigen R (HuR), tristetraprolin (TTP), ubiquitin specific peptidase 10 (USP10), and T-cell intracellular antigen-1 (TIA-1) [17–20]. Among them, Ras-GAP SH3 domain binding protein 1 (G3BP1) and Ras-GAP SH3 domain binding protein 2 (G3BP2) are critical to SG formation. And more importantly—as key components of SGs—they are also critical to the functions performed by SGs [21–23].

#### The initiation of SGs

In addition to the composition of SGs, paying attention to the molecular mechanism underlying the formation of SGs is essential for targeting SGs as an antitumor strategy. SGs are formed under various cellular stress conditions such as oxidative, osmotic, heat-shock, UV irradiation, and proteotoxic stress [12, 13, 24, 25]. Furthermore, pathogens such as *E. coli* also contribute to the formation of SGs. Indeed, Tsutsuki et al. have reported that the Subtilase cytotoxin produced by *E. coli* can promote the formation of SGs [26]. Previous studies have shown that SGs are formed via mRNA stagnation at the initiation of translation [12–16, 27]. Specifically, the stress-induced phosphorylation of eukaryotic initiation factor 2α (eIF2α) leads to mRNA translation stagnation and subsequent SGs formation. [12, 18]. The “Liquid–Liquid Phase Separation (LLPS) First” and the “Cores First” models are typical explanations for the SG assembly process [12, 28, 29]. One study found that Lsm7 liquid-liquid phase separation triggers the formation of SGs [30], while others reported that the core protein-RNA interaction network that encodes the SG formation. Long, single-stranded, unfolded RNA induces a conformational conversion of G3BP, which is necessary for SG formation. Furthermore, G3BP1 acts as the central node and tunable switch of the network that can assemble SGs by triggering phase separation [20, 31]. Ripin et al. reported that RNA aggregation is a key step in the formation of SGs, which is analogous to the formation of misfolded protein aggregates. In other words, SGs are the RNA equivalent of misfolded protein aggregates. These authors also suggested that intramolecular RNA-RNA interactions may be the driving force for RNA aggregation. When RNA aggregation exceeds the RNA chaperone’s ability to break down RNA, several human diseases can ensue [32].

As SG formation is strongly associated with cancer, herein, we review recent research and elucidate the mechanisms that promote the formation of SGs in tumor cell. Mammalian target of rapamycin (mTOR) is a key signaling molecule that regulates cellular biological behaviors such as survival, proliferation, and metabolism [33, 34]. Based on the regulatory role of mTOR in cell biological behavior, it follows that mTOR plays an important role in tumorigenesis and tumor development [34]. Numerous studies have found that upregulation of mTOR promotes SG formation in cancer cell, which may be mediated by eukaryotic translation initiation factor 4E binding protein 1 (EIF4EBP1) and the ribosomal proteins S6 kinase 1 and 2 (S6K1, S6K2) [35–38]. In addition, mutations of the RAS genes (KRAS, NRAS, and HRAS), as well as MG53, contribute to the formation of SGs [39–41]. These RAS genes constitute some of the most commonly mutated genes in tumor cell, with mutations in RAS genes detected in approximately 30% of all tumors [42]. Further research found that KRAS mutation promotes the formation of SGs in pancreatic cancer by regulating the biosynthesis and catabolism of the lipid signaling molecule 15-deoxy-Δ^12,14^-prostaglandin J_2_ (15d-PGJ2) [43]. MG53 can promote the formation of SGs in non-small cell lung cancer by regulating the activity of G3BP2 [41]. The overexpression of histone deacetylase (HDAC) proteins has been shown to promote SG formation by deacetylating G3BP1 or interacting with dynamin and microtubules [44, 45]. Furthermore, the RNA-binding protein musashi-1 (MSI1) promotes the formation of SGs in colorectal cancer cell [46]. MSI1 promotes SGs formation via the PKR/eIF2α signaling pathway, thereby contributing to chemotherapy resistance [47].

These findings demonstrate that the formation of SGs is the result of the multifaceted regulation of multiple pathways. Further research will unravel additional novel mechanisms, which will help to facilitate the development of strategies to block the formation of SGs and inhibit cancer progression.

#### The disassembly of SGs

In opposition to SG formation, SGs disintegrate when external stress subsides [12, 48, 49]. Similar to SG assembly, SG disassembly is a multi-step process wherein the unstable shell is first dissolved, followed by core structure disassembly. The larger core structure breaks into smaller foci cleared via autophagy [48]. Moreover, studies have confirmed that the depolymerization of SGs is associated with molecular chaperones such as heat shock protein 70 (Hsp70). A possible mechanism for this depolymerization is that molecular chaperones promote SG disintegration by inhibiting the accumulation of misfolded proteins in SGs [48, 50]. Helicase is also involved in SGs disassembly; for instance, RNA/DNA helicases, using energy from ATP hydrolysis, displace proteins bound to nucleic acids or unlock DNA/RNA, thereby regulating SGs disassembly [11]. However—despite these discoveries—there remain many unknowns regarding the depolymerization of SGs; therefore, further investigation is warranted.

#### The function of SGs

A previous study has determined that SGs store and target mRNA for degradation under stress [51]. However, further research has provided deeper insights into the functions of SGs, such as the regulation of several physiological activities [52]. SGs contain catalytic and signaling proteins, hence excessive formation of SGs has a critical impact on cell metabolism and survival [52]. Until now, it has been widely assumed that the mRNA contained in SGs is not translated into protein, imparting that SGs mediate translation inhibition under stress [53–55]. However, a recent study showed that SGs not only contain untranslated mRNA but that some of the mRNA could be translated into proteins, with certain mRNA molecules undergoing complete translation cycles [25]. This discovery has challenged conventional wisdom, further demonstrating that SGs can participate in and regulate various cellular biological processes.

Numerous studies have explored the function of SGs, many of which have implicated them in the occurrence and progression of a multitude of diseases by modulating various signaling pathways [9, 56–58]. While acute and chronic stress particles have different effects on human diseases [49], SG formation has been closely linked to the occurrence of diseases associated with inflammatory conditions and stress [5]. In stressed cell, SG formation leads to the isolation of DDX3X, which inhibits DDX3X-mediated activation of the NLRP3 inflammasome and ultimately inhibits programmed inflammatory cell necrosis [59]. SGs share considerable protein with neuron particles, rendering them closely related to degenerative diseases and multisystem proteinopathy [57, 60–62]. For example, as components of SGs, ATX1, hnRNPA1, TDP-43, TIA1, and TAF15 can lead to the occurrence of neurodegenerative diseases such as Alzheimer’s disease (AD), amyotrophic lateral sclerosis (ALS), and frontotemporal dementia (FTD) [63, 64]. SGs are also associated with viral infection, aging, cerebral ischemia, atrial fibrillation, and organ fibrosis [65–68]. Intriguingly, SGs also take part in the occurrence and development of various cancers [69].

### The functional differences between SGs in normal cell biology and cancer cell pathology

In adverse conditions, the formation of SGs in normal cell can protect cell from damage and promote cell survival [24, 70, 71]. In response to environmental stress, SGs promote the transcriptional activation of mRNA encoding proteins essential for stress responses, such as promoting HSP70 expression during heat shock [72]. Previous studies have also found that SGs may play a role as a signal hub, regulating cell metabolism and promoting survival by recruiting signal protein [73]. These findings suggest SGs as an important strategy to resist various stress injuries and maintain cell survival in normal cell. However, the mechanism underlying the anti-stress role of SGs in normal cell remains unclear. Notably, when stress persists, excessive formation of SGs can lead to pathological aggregation of SGs, which can promote the occurrence of various diseases, including various tumors (such as breast, lung, and prostate cancers) [41, 74–76]. The roles and mechanisms of SGs in cancer cell pathology will be described extensively in the following paragraphs.

### Role of SGs in cancer

**Fig. 2:**
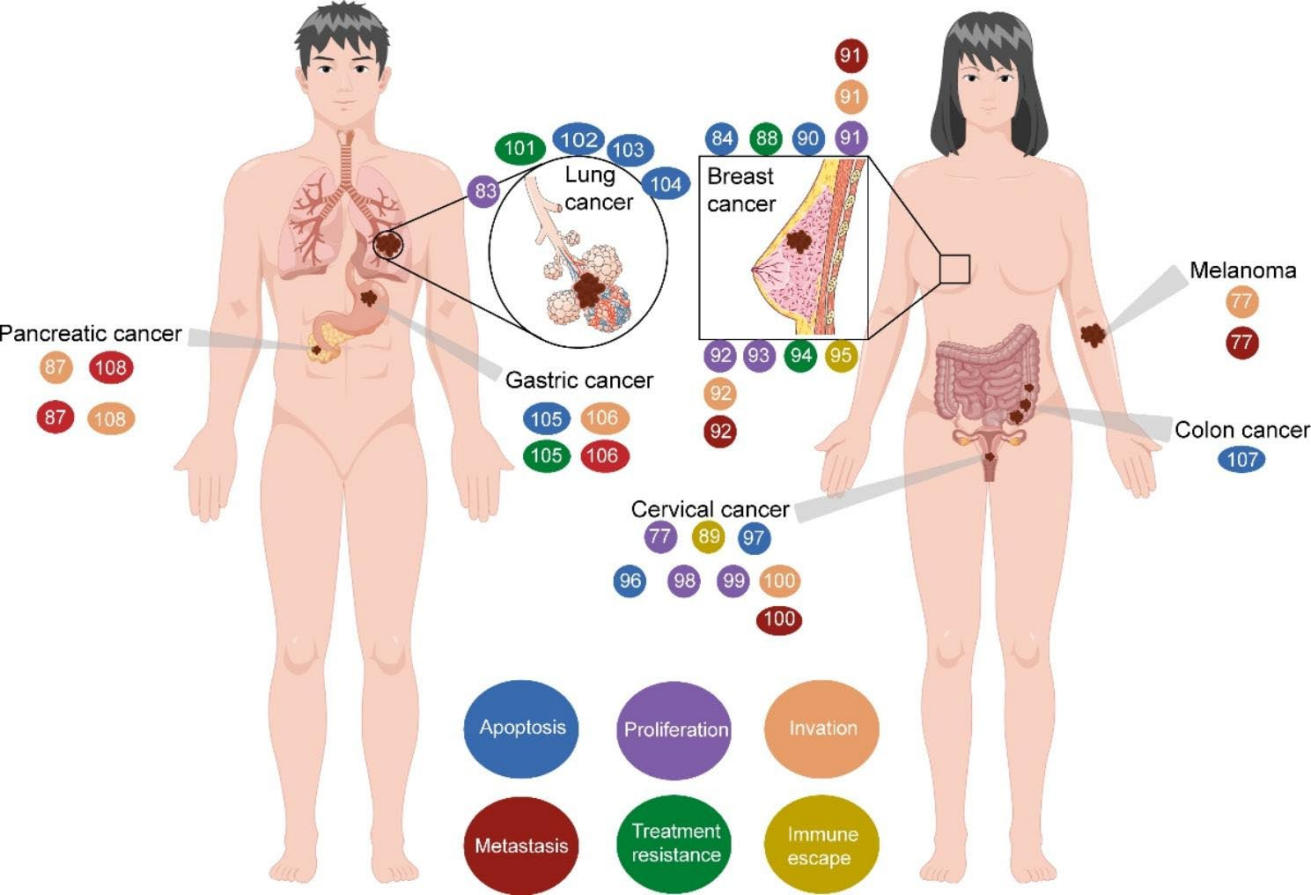
**Role of SGs in cancer.** SGs play a critical role in the regulation of cell apoptosis, cell proliferation, cell invasion and metastasis, and therapy resistance in cancer. The numbers indicate references

SGs play an important role in the occurrence and development of tumors [5–7, 17, 77]. Stress adaptation is an increasingly important characteristic of cancer cell [55, 78]. Compared with normal cell, SG-related components are upregulated in various tumor cell, including G3BP1 and G3BP2 [8, 79, 80]. In addition, high SG expression levels contribute to poor outcomes in cancer patients [81, 82]. Evidence indicates that cancer cell may generate SGs to protect expressed mRNAs—which regulate cell metabolism, signal transduction pathways, and stress responses and promote their own survival, transfer, and other biological behavior—from degradation [16, 24, 55, 74]. At present, there is sufficient evidence that SGs play a key role in promoting tumor cell proliferation and inhibiting tumor cell apoptosis [83–85]. SGs have also been found to promote the invasion and migration of tumor cell, thereby promoting tumor progression [86, 87]. Moreover, SGs are an important factor in tumor treatment resistance, including chemotherapy and radiotherapy resistance, which contributes to poor clinical treatment efficacy for tumor patients [84, 88]. SGs also regulate the tumor immune microenvironment (TIME), and lead to immune escape in tumor cell [89]. The following sections discusses the role of SGs in regulating these cellular processes and the molecular mechanisms involved (Table 1; Fig. 2).

**Table 1:** SG-related signaling pathways in cancer

Cancer type	Downstream	Expression	Functions	References
Breast cancer	JNK	↓	Inhibits apoptosis	[84, 90]
	β-catenin	↑	Promotes proliferation, invasion and metastasis	[91, 92]
	MYC	↑	Promotes invasion and metastasis	[92]
	PMP22	↓	Promotes proliferation	[93]
	LINE-1	↑	Promotes chemotherapeutic resistance	[94]
	HIF-1α	↑	Promotes radiation resistance	[88]
	PD-L1	↑	Promotes immune escape	[95]
	mTORC1	↓	Inhibits apoptosis	[96]
Cervical cancer	ROS	↓	Inhibits apoptosis	[97]
	RBFOX2	↑	Promotes proliferation	[77, 98, 99]
	RNH1	↓	Promotes invasion and metastasis	[100]
	PD-1	↑	Promotes immune escape	[89]
	ROS	↓	Promotes radiation resistance	[101]
Lung cancer	p53	↓	Inhibits apoptosis	[102–104]
	LET-7	↓	Promotes proliferation	[83]
	YWHAZ	↑	Inhibits apoptosis, promotes chemotherapeutic	[105]
Gastric cancer			resistance	
	TGF -β	↑	Promotes invasion and metastasis	[106]
	β-catenin	↑	Inhibit apoptosis	[107]
Colon cancer				
Pancreatic cancer	BART	↑	Promotes invasion and metastasis	[87, 108]
Melanoma	RB1	↑	Promotes invasion and metastasis	[77]

#### SGs inhibit apoptosis of tumor cell

**Fig. 3:**
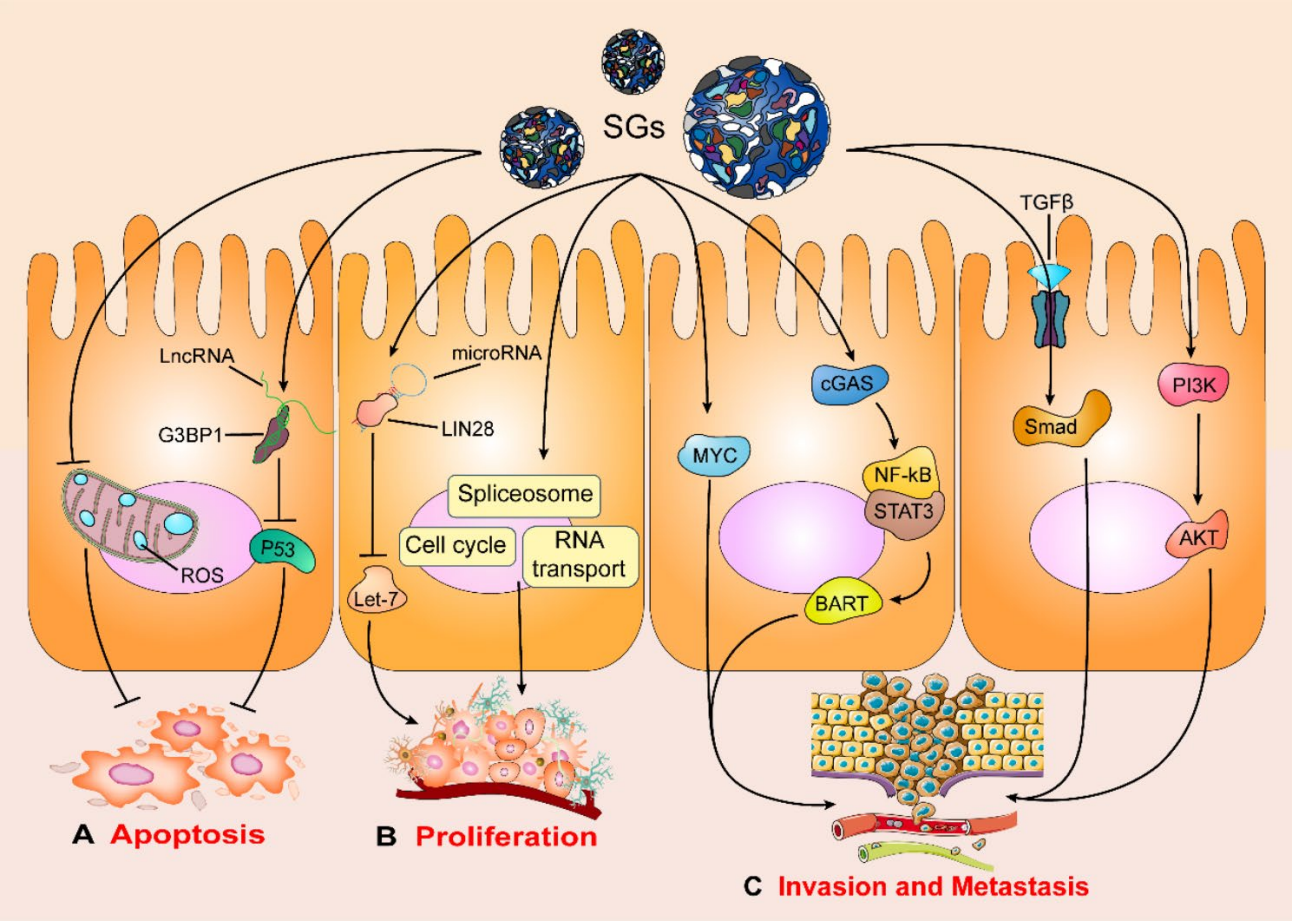
**SGs regulate cell apoptosis, cell proliferation, and cell invasion and metastasis in cancer.** SGs **A** inhibit tumor cell apoptosis by regulating p53, and ROS, **B** promote tumor cell proliferation by regulating LET-7, spliceosome, RNA transport, and cell cycle, and **C** promote tumor cell invasion and metastasis by regulating MYC, BART, TGF-β, and AKT.

The effect of SGs on cell apoptosis may be their earliest explored function, with evidence supporting their critical role in regulating tumor cell apoptosis (Fig. 3A).

Previous findings have indicated that SGs participate in the MAPK [85], mTORC1 [90], ROS [91], and Wnt/β-catenin [92] pathways to inhibit tumor cell apoptosis. More than a decade ago, it was reported that SGs inhibit apoptosis by regulating the MAPK pathway, including the classic JNK and p53 pathways [85]. One such example is the inhibition of JNK activation and JNK-mediated breast cancer cell apoptosis by SGs in recruiting rho-associated, coiled-coil-containing protein kinase 1 (ROCK1) to prevent the phosphorylation of JNK-interacting protein 3 (JIP3) [84, 93]. In lung cancer, G3BP1 has been shown to inhibit cell apoptosis by negatively regulating the p53 tumor suppressor gene [94–96]. For instance, Chao et al. found that G3BP1 interacts with lncRNA to promote lung cancer-cell apoptosis through nuclear sequestration of p53 [96]. Furthermore, the mammalian target of rapamycin complex 1 (mTORC1) pathway is another key mechanism by which SGs inhibit cervical cancer cell apoptosis [90] via regulation of the eIF4F complex assembly and TIA1/TIAR protein recruitment in SGs [17, 97, 98]. SGs have also been shown to inhibit cervical cancer cell apoptosis by inhibiting ROS production, which may be mediated by the activation of ubiquitin-specific peptidase 10 (USP10) [91]. Both vitro and vivo studies demonstrated that G3BP1 inhibits apoptosis in colon cancer cell and promotes colon cancer progression by activating the β-catenin signaling pathway [92].

Isolation of the receptor for activated C kinase-1 (RACK1)—a known pro-apoptotic factor—within SGs inhibits tumor cell apoptosis. RACK1 isolation in SGs reduces caspase-3 activity—a possible mechanism for apoptosis inhibition [99]. Further studies found that isolation of RACK1 into SGs negatively affected the stress-activated P38/JNK pathway, thus inhibiting apoptosis [100]. Moreover, recent evidence suggests that G3BP1 inhibits gastric cancer cell apoptosis via the YWHAZ/Bax axis [101].

#### SGs promote tumor cell proliferation

A number of in vitro and vivo studies have suggested that promoting tumor cell proliferation is an important mechanism whereby SGs promote tumor progression. In this regard, scientists have achieved substantial results (Fig. 3B). SGs recruit cell cycle-related mRNA through RBFOX2, including RB1, ABL2, PDGFRA, and GSK3B mRNAs, thereby promoting cervical cancer cell proliferation [77, 102, 103]. Moreover, recruiting and promoting the expression of adenylate-uridylate-rich elements (AU-rich elements; AREs) is another mechanism by which SGs promote tumor cell proliferation [103].

In addition to SGs, certain SG components individually affect tumor cell proliferation. For example, while HuR—also known as ELAV-like protein 1 (ELAV1)—and TTP are RNA-binding proteins integrated as part of SGs, they both promote tumor cell proliferation in and of themselves. TTP is involved in the promotion of MYC-mediated tumor proliferation [8, 104], while HuR overexpression increases tumor size and weight in mice, which is related to HuR’s regulation of the spliceosome, RNA transport, and the cell cycle [105–107]. LIN28—a versatile RBP—is another component of SGs. LIN28-mediated downregulation of LET-7 microRNAs upregulates LET-7 expression, which promotes lung cancer cell proliferation [83]. G3BP1, a key component protein of SGs, binds to specific RNA molecules through its C-terminal RNA recognition motif (RRM) to regulate the stability of mRNA and affect the proliferation of tumor cell [108]. A case in point is G3BP1’s degradation of peripheral myelin protein 22 (PMP22) mRNA to inhibit the expression of PMP22, which can promote the proliferation of breast cancer cell [109]. G3BP1 can also inhibit the phosphorylation and degradation of β-catenin and promote the proliferation of human breast cancer cell via interaction with GSK-3β [110]. In summary, these results suggest that SGs regulate cell proliferation in various cancer.

#### SGs regulate cancer invasion and metastasis

Invasion and metastasis have posed major challenges in the clinical treatment of cancer patients, constituting the main cause of death in most cases. Notably, increasing evidence indicates that SGs regulate a variety of signaling pathways to promote the invasion and metastasis of tumor cell (Fig. 3C).

G3BP transcriptionally regulates the expression of ARL2 (BART), ultimately promoting the invasion and metastasis of pancreatic cancer cell [87, 111]. SGs mediate cervical cancer cell metastasis by inhibiting ribonuclease inhibitor 1 (RNH1) to promote angiopoietin activity [112], while tudor domain containing 3 (TDRD3) located within SGs is one of the key factors promoting breast cancer cell invasion and lung metastasis. Further studies have found that TDRD3 regulates the translation of key genes such as MYC and β-catenin [113]. In human sarcoma, the Y-box binding protein 1 (YB1) enhances the formation of SGs by binding directly to G3BP1’s 5’UTR and promoting G3BP1 mRNA translation, thus enhancing the invasion and metastasis of human sarcoma cell [86]. In gastric cancer cell, epidermal growth factor (EGF) regulates the expression of ATXN2L through the PI3K/Akt signaling pathway, thereby increasing the formation of SGs [114]. Furthermore, SGs promote the activation of the TGF-β/Smad signaling pathway, ultimately promoting the invasion and metastasis of gastric cancer cell [115]. In esophageal cancer, increased G3BP1 expression enhances the migration and invasion ability of esophageal cancer cell by activating the Wnt/β-catenin and PI3K/AKT signaling pathways [116]. In melanoma, SGs mediate RNA binding fox-1 homolog 2 (RBFOX2) localization and further promote retinoblastoma 1 (RB1) protein levels along with mRNA expression, thus significantly promoting melanoma cell metastasis and tumor growth [77]. In age-related tumors, G3BP1 activates the NF-κB and STAT3 pathways via cyclic GMP-AMP synthase (cGAS), promoting the senescence-associated secretory phenotype (SASP) and stimulating the migration of tumor cell [117].

However, the role of SGs in other tumors remains unknown. Further research is, therefore, necessary to further connect SGs with malignancies to better tackle the problem of distant metastasis of tumors.

#### SGs mediate radiation resistance

**Fig. 4:**
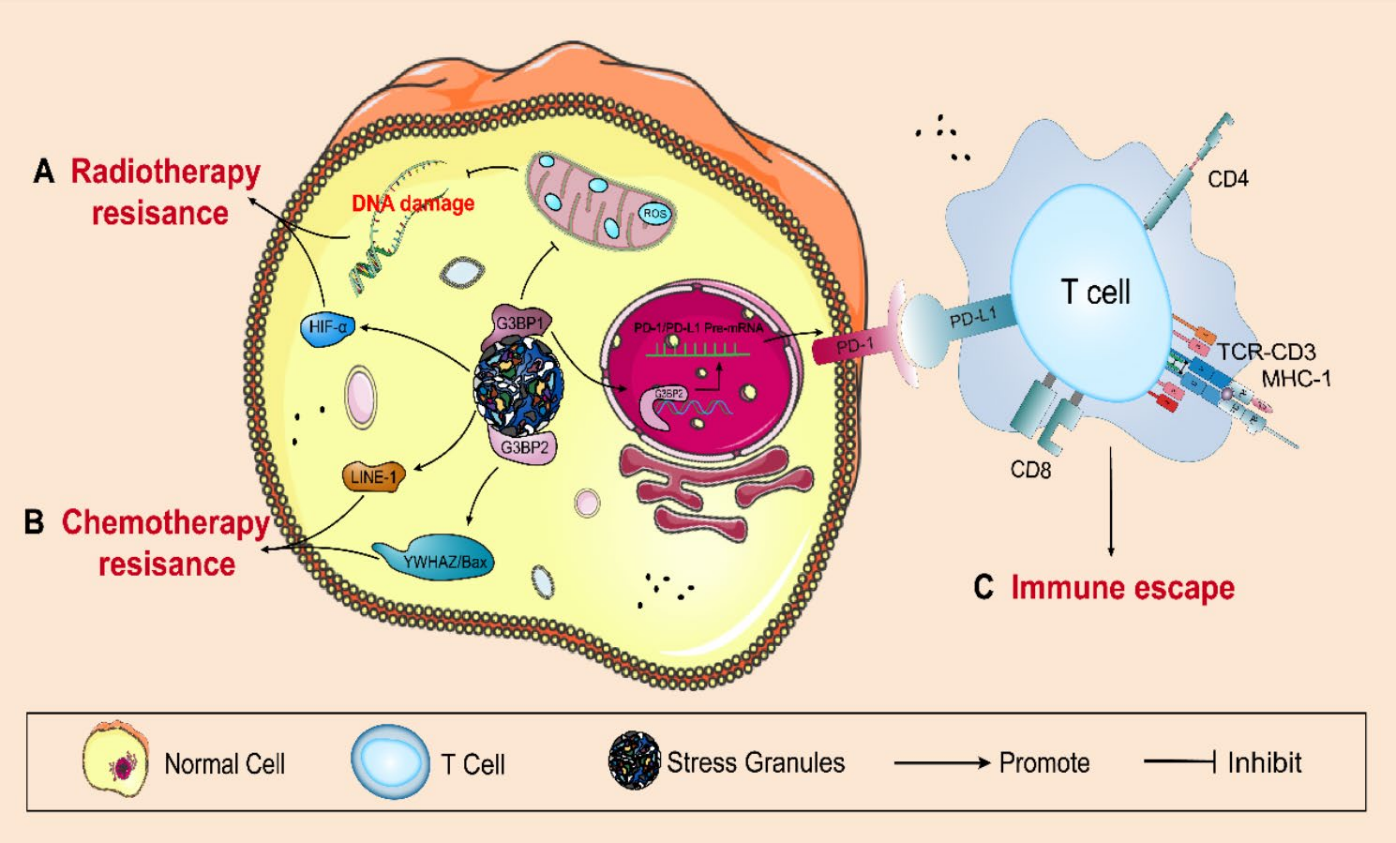
**SGs promote chemotherapy resistance, radiation resistance, and immune escape.** SGs **A** promote radiation resistance by regulating ROS and HIF-α, **B** promote chemotherapy resistance by upregulating the expression of LINE-1 and YWHAZ/Bax, and **C** regulate PD-1 and PD-L1 to facilitate immune escape

Mediating radiotherapy resistance is another important function of SGs. Radiotherapy can increase the formation of SGs in cancer cell [16]. In turn, excessive formation of SGs is a major cause of radiation resistance in tumor cell [17, 88]. Several SG-mediated radiation resistance pathways have been identified (Fig. 4B). SGs promote the tolerance of breast cancer cell to radiotherapy by regulating HIF-1α [88]. Recent evidence suggests that knockdown of G3BP in lung cancer can damage the ROS clearance system, contributing to increased radiosensitivity of cell. In other words, increased formation of G3BP1 reduces ROS production and inhibits radiation-induced DNA damage and apoptosis, leading to radiotherapy resistance in non-small cell lung cancer (NSCLC) cell [118].

Data on the effects of SGs on tumor cell radiation resistance are limited; therefore, further investigation into the mechanisms involved is necessary.

#### SGs induce chemotherapy resistance

Similar to radiotherapy resistance, SGs are also involved in chemotherapy resistance. Chemotherapy resistance is a key problem in tumor therapy with a complicated molecular mechanism [119]. It has been reported that anticancer drugs induce the assembly of SGs in tumor, while an increase in SGs formation promotes the resistance of tumor cell to various anticancer drugs [8, 16, 52, 120].

In theory, any chemotherapeutic agent that affects the translational process or targets translational elements could lead to SG assembly [121]. Currently, it is widely understood that chemotherapeutic agents induce SGs formation by promoting the phosphorylation of eukaryotic initiation factor 2α (eIF2α). However, different chemotherapies induce different mechanisms of eIF2α phosphorylation. Adjibade et al. found that the endoplasmic reticulum (ER) stress induced by sorafenib activates PKR-like endoplasmic reticulum kinase (PERK), thereby promoting the phosphorylation of eIF2α and the formation of SGs, a leading cause of sorafenib drug resistance [120]. Interestingly, lapatinib has also been shown to promote the phosphorylation of eIF2α by activating PERK [122]. Similarly, 5-FU activates protein kinase PKR, leading to the phosphorylation of eIF2α, thereby facilitating the *de novo* assembly of SGs [123]. Bortezomib activates eIF2α kinase HRI to trigger eIF2α phosphorylation, which promotes SG formation [124].

However, mediating eIF2α phosphorylation is not the only way for chemotherapy drugs to induce SG formation. For example, the vinca alkaloid (VA) class of anti-neoplastic agents inhibits mTOR and activates eIF4EBP1 to destroy the eIF4F complex, thereby facilitating SGs assembly [119].

SGs can induce chemotherapy resistance in tumor cell [40, 125, 126]. For instance, studies have shown that inhibiting the formation of SGs induced by hypoxia can enhance sensitivity to cisplatin and paclitaxel in human cervical cancer cell [125]. In vitro and vivo findings have shown that SPOP mutations promote SGs assembly by inhibiting caprin1 ubiquitination and degradation, ultimately mediating docetaxel resistance in prostate cancer [76]. In various tumor cell, psammaplysin F inhibits the formation of SGs, thereby increasing the efficacy of bortezomib and sorafenib [126]. Notably, we have previously reported that upregulation of the SGs regulator ATXN2L promotes SGs assembly, thus reducing the sensitivity of gastric cancer cell to oxaliplatin [114].

However, the specific molecular mechanism underlying the capacity of SGs to mediate chemotherapy resistance remains unknown. Next, we will describe several possible molecular mechanisms identified so far (Fig. 4A). One study found that G3BP1 interacts with YWHAZ to isolate Bax in the cytoplasm, thereby enhancing chemotherapy resistance in gastric cancer [101]. Meanwhile, another study determined that the formation of SGs induced by paclitaxel can increase the expression of long interspersed element-1 (LINE-1), thereby rendering triple-negative breast cancer cell resistant to chemotherapy [127].

These findings suggest that inhibiting SGs assembly and/or blocking SGs mediation of signaling pathways related to drug resistance can reverse chemotherapy resistance in various tumor cell.

#### SGs participate tumor immunity

SGs not only regulate tumor cell proliferation, apoptosis, and invasion and metastasis—as well as chemotherapy and radiotherapy resistance—but also play a critical role in controlling tumor immunity (Fig. 4C). Novel research regarding human immune checkpoints has identified that SGs can regulate immune checkpoint molecules such as programmed death-1 (PD-1) and programmed death-ligand 1 (PD-L1) to help tumor cell to suppress and evade the immune system [89, 128]. For example, microtubule targeting drugs (MTDs) inhibit PD-1 expression in cancer therapy, which is related to SGs assembly in that microtubules and molecular motor kinase 1 are critical for this SG-dependent regulation [89]. Moreover, Zhang et al. discovered that PD-L1 protein levels in breast cancer cell increased under stress conditions and were dependent on G3BP2. G3BP2 may stabilize PD-L1 mRNA via the RRM domain, thereby promoting PD-L1 levels. The authors also found that the small molecule C108—upon binding to G3BP2—reduced PD-L1 expression by enhancing mRNA degradation and promoted tumor immune cell infiltration in tumor-bearing mice [128]. Although the link between tumor immunity and SGs remains uncertain, these results provide ample evidence that targeting SGs may offer tumor immunotherapy benefits as well as an unexpected impact on immunotherapy protocols.

### Cancer therapy based on targeting SGs

**Fig. 5:**
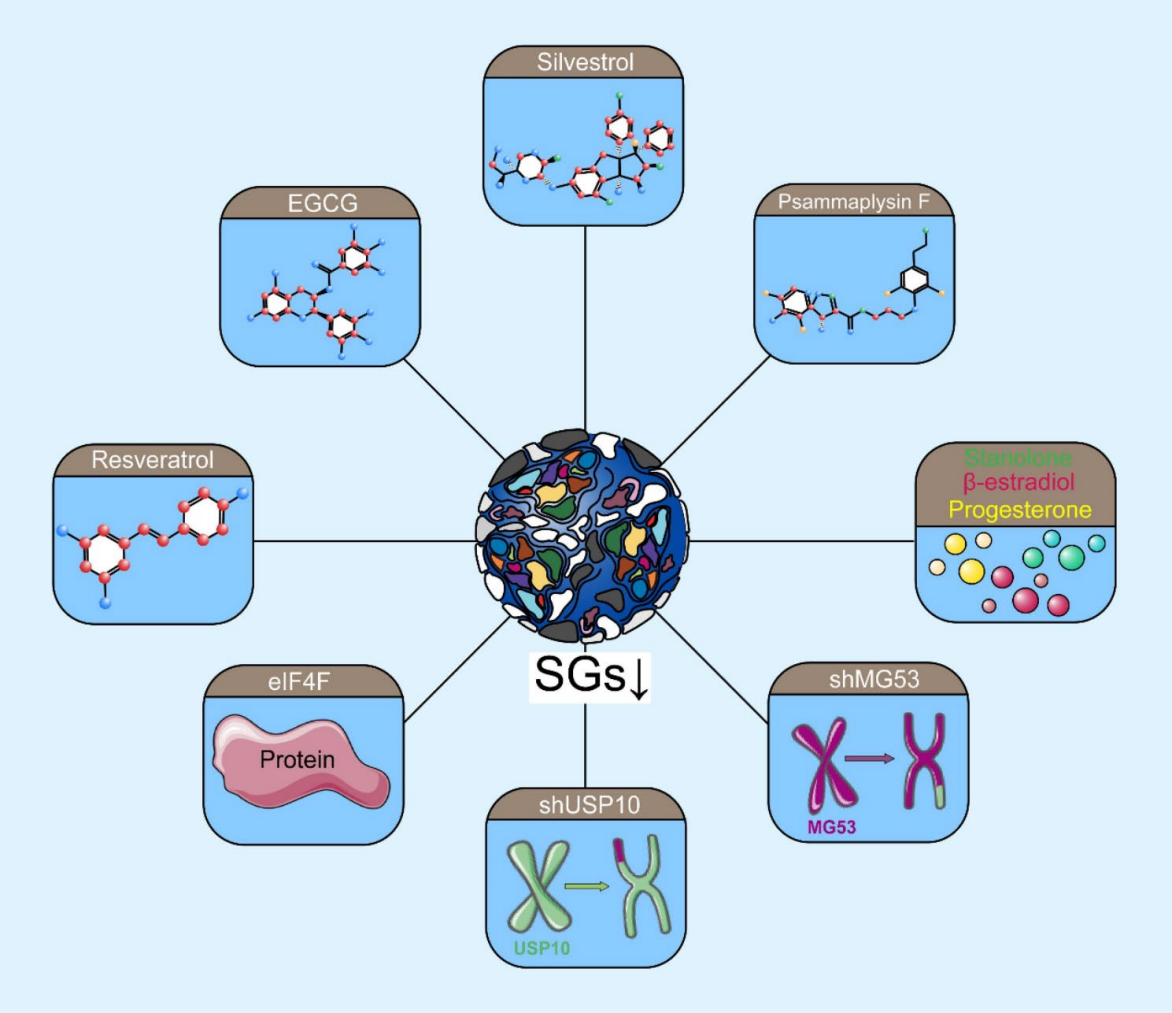
**Targeting SGs as a cancer treatment strategy.** Targeting G3BP1/G3BP2 and eIF families or using SGs inhibitors (such as psammaplysin F and silvestrol) can inhibit SG formation, thereby improving cancer treatment efficacy and outcome

At present, several studies have confirmed that inhibiting SG recruitment or microtubule aggregation, as well as other strategies that prevent SG formation, inhibits the occurrence and development of tumors [69]. In view of the plethora of existing research results surrounding SGs, we summarize the following methods to target SGs for cancer treatment (Fig. 5).

It has been reported that high expression levels of SG components suggest poor prognosis in cancer [87, 129–133]. Targeting SGs is a potentially important therapeutic strategy against cancer, especially by targeting G3BP1/G3BP2. In an vivo study, inhibiting G3BP2 activity by knocking down MG53 significantly reduced tumor volume and weight in mouse lung cancer model [41], which suggests that targeting G3BP2 had a significant anticancer effect in NSCLC. Other studies have determined that USP10 knockdown can inhibit G3BP2 formation, thereby inhibiting the growth of prostate cancer cell [81]. Silencing G3BP1 expression in vivo can inhibit breast cancer cellcell migration, whereas G3BP1 knockout in mice can inhibit distant metastasis of tumors [134]. Interestingly, researchers have identified two anti-tumor drugs targeting G3BP1—resveratrol and epigallocatechin gallate (EGCG). Resveratrol not only promotes tumor cell apoptosis and inhibits tumor cell proliferation, but can also be used as a preventive drug against melanoma [135–137]. Recent studies have found that the anti-tumor effect of resveratrol may be realized by targeting G3BP1 and leading to increased p53 expression [95, 138]. Similar to resveratrol, EGCG has significant antitumor properties [139–142]. Further studies confirmed that EGCG inhibits Ras activation by blocking the interaction between G3BP1 and RAS-GAP, which ultimately plays an anticancer role in lung cancer [143].

The eIF family is closely related to the formation of SGs; thus, targeting these factors can significantly improve the therapeutic effects of tumor treatments. Previous studies have found that eIF2α is a critical translation initiation factor of SG formation. The phosphorylation of eIF2α is a key step that triggers the formation of SGs [12]. Kimberley et al. found that psammaplysin F—a natural product isolated from a marine sponge—can inhibit the formation of SGs by inhibiting the phosphorylation of eIF2α. This treatment—when used in conjunction with bortezomib and sorafenib—might enhance the therapeutic effect in cervical cancer [126, 144]. Vilas-Boas et al. found that inhibiting eIF2α phosphorylation also renders glioma cell sensitive to chemotherapy [145]. In addition to eIF2α, the eIF4F complex also regulates the formation of SGs [146]. Targeting eIF4F complexes may inhibit SG formation and tumor progression in human breast and prostate cancers [147].

The use of SGs inhibitors is also an effective way to promote the efficacy of cancer treatment. Shikshya et al. found that chemical compounds including β-estradiol, progesterone, and stanolone can disrupt the formation of SGs in HeLa cell, thereby improving the efficacy of chemotherapy for cervical cancer [125]. Kimberley et al. found that psammaplysin F inhibits the formation of SGs, which can enhance the therapeutic effect of cervical cancer [126, 144]. Similarly, silvestrol, a flavin derivative, affects the formation of SGs to inhibit tumor growth in human breast and prostate cancer xenograft mice [147].

These results suggest targeting SGs as a promising cancer treatment strategy that can significantly improve clinical outcomes and the quality of life in cancer patients.

## Conclusions and future perspectives

SGs are non-enveloped structures formed by cell under stressful conditions that help cell to cope with stress-related damage and promote autotrophic survival [5]. SGs have been widely detected in tumor cell and therefore become a prime characteristic of malignant cell [55, 78]. Stress-induced eIF2α phosphorylation leads to mRNA translation stagnation and subsequent SGs formation [12, 18]. In addition, several studies have determined that cancer-related gene mutations and abnormal signaling pathways in cancer induce SGs formation. For example, evidence showed that the upregulation of mTOR, the mutation of RAS gene, and MG53 promote the formation of SGs [35–41]. However, the mechanisms involved are yet to be fully elucidated. As emerging cancer-promoting substances, SGs play an important role in the genesis and development of tumors [130, 148]. In this review, we delved into the latest research on SGs in cancer and summarized the mechanisms by which SGs mediate tumor cell proliferation, apoptosis, invasion and metastasis, chemotherapy resistance, radiotherapy resistance and immune escape. However, the nature of these roles and mechanisms remains inconclusive. In addition to the roles mentioned above, any potential involvement of SGs in the regulation of tumor cell metabolism (such as glucose and fatty acid metabolism), tumor cell dryness, epithelial-mesenchymal transition (EMT), and other biological processes should be further studied to clarify and enrich our understanding of the mechanisms associated with SGs.

Although the roles and mechanisms of SGs in tumorigenesis and tumor development require further study, the current findings are sufficient to demonstrate that SGs are a key regulatory hub and a promising therapeutic target against cancer. Previous studies have confirmed that targeting key components of SGs (such as G3BP1/2, eIF2α, and eIF4F complex) can enhance therapeutic efficacy [41, 134, 145, 146]. In addition, SGs inhibitors psammaplysin F and silvestrol have been found to inhibit tumor growth and promote the effect of chemotherapy [126, 144, 147]. As such, they provide a reliable reference for the development of new cancer treatment methods. Existing results suggest that targeting the upstream and downstream pathways of SGs may contribute to the efficacy of therapeutic interventions for cancer patients. Targeting SGs may be an effective strategy to mitigate drug resistance in cancer treatment. However, additional research remains warranted prior to clinical applications. First, it is difficult to determine whether the roles and associated signaling pathways of SGs are specific to certain cancers with unique characteristics or are generally applicable to most cancers. A deeper understanding of SG mechanisms will clarify this matter. Second, SGs are associated with several other diseases, besides cancer, such as viral infections, neurodegenerative diseases, and cardiovascular diseases [149, 150]. Methods to specifically inhibit the formation of SGs in tumor cell are therefore difficult to develop. Finally, data on the biomarkers associated with SGs are limited; as such, it is critical to investigate these biomarkers.

The use of new, advanced sequencing technologies will help to elucidate the roles and mechanisms of SGs in tumors and ultimately accelerate the clinical application of SGs in diagnosis, treatment, and prognosis assessments. Ideally, targeting SGs may be used as a novel anti-tumor strategy in the near future.

## Data Availability

Not applicable.

## Abbreviations

SGs: Stress granules
RNP: Ribonucleoprotein
RBPs: RNA binding proteins
eIF3: Eukaryotic initiation factor 3
eIF4G: Eukaryotic initiation factor 4G
eIF4A: eukaryotic initiation factor 4 A
G3BP: Ras-GAP SH3 domain binding protein
PABP: Poly(A)-binding protein
CAPRIN1: Cell cycle associated protein 1
UBAP2L: Ubiquitin associated protein 2 like
HuR: Human antigen R
TTP: Tristetraprolin
USP10: Ubiquitin specific peptidase 10
TIA-1: T-cell intracellular antigen-1
eIF2: eukaryotic initiation factor 2
mTOR: Mammalian target of rapamycin
EIF4EBP1: Eukaryotic translation initiation factor 4E binding protein 1
S6K1, S6K2: S6 kinase 1 and 2
15d-PGJ2: 15-deoxy-Δ^12,14^-prostaglandin J_2_
HDAC: Histone deacetylase
MSI1: Musashi-1
Hsp70: Heat shock protein 70
AD: Alzheimer’s disease
ALS: Amyotrophic lateral sclerosis
FTD: Frontotemporal dementia
TIME: Tumor immune microenvironment
ROCK1: Rho-associated, coiled-coil-containing protein kinase 1
JIP3: JNK-interacting protein 3
mTORC1: Mammalian target of rapamycin complex 1
RACK1: Receptor for activated C kinase-1
AREs: Adenylate-uridylate-rich elements (AU-rich elements)
ELAV1: ELAV-like protein 1
RRM: RNA recognition motif
PMP22: Peripheral myelin protein 22
RNH1: Ribonuclease inhibitor 1
TDRD3: Tudor domain containing 3
YB1: Y-box binding protein 1
EGF: Epidermal growth factor
RBFOX2: RNA binding fox-1 homolog 2
RB1: Retinoblastoma 1
cGAS: Cyclic GMP-AMP synthase
SASP: Senescence-associated secretory phenotype
ER: Endoplasmic reticulum
eIF2α: Eukaryotic initiation factor 2α
PERK: PKR-like endoplasmic reticulum kinase
VA: Vinca alkaloid
LINE-1: Long interspersed element-1
NSCLC: Non-small cell lung cancer
PD-1: Programmed death-1
PD-L1: Programmed death-ligand 1
MTDs: Microtubule targeting drugs
EGCG: Epigallocatechin gallate
EMT: Epithelial-mesenchymal transition
